# Developing Transparent and Conductive PolyHEMA Gels Using Deep Eutectic Solvents

**DOI:** 10.3390/polym15122605

**Published:** 2023-06-08

**Authors:** Tai-Yu Chen, Yi-Jie Jiang, Hsiu-Wen Chien

**Affiliations:** 1 Department of Chemical and Materials Engineering, National Kaohsiung University of Science and Technology, Kaohsiung 807618, Taiwan; terry25197@gmail.com (T.-Y.C.); c108146230@nkust.edu.tw (Y.-J.J.); 2 Photo-Sensitive Material Advanced Research and Technology Center (Photo-SMART Center), National Kaohsiung University of Science and Technology, Kaohsiung 807618, Taiwan

**Keywords:** HEMA, DES gels, hydrogels, dehydration, conductivity

## Abstract

Poly(2-hydroxyethyl methacrylate) (polyHEMA) hydrogels are commonly used in biomaterials such as contact lenses. However, water evaporation from these hydrogels can cause discomfort to wearers, and the bulk polymerization method used to synthesize them often results in heterogeneous microstructures, reducing their optical properties and elasticity. In this study, we synthesized polyHEMA gels using a deep eutectic solvent (DES) instead of water and compared their properties to traditional hydrogels. Fourier-transform infrared spectroscopy (FTIR) showed that HEMA conversion in DES was faster than in water. DES gels also demonstrated higher transparency, toughness, and conductivity, along with lower dehydration, than hydrogels. The compressive and tensile modulus values of DES gels increased with HEMA concentration. A DES gel with 45% HEMA showed excellent compression–relaxation cycles and had the highest strain at break value in the tensile test. Our findings suggest that DES is a promising alternative to water for synthesizing contact lenses with improved optical and mechanical properties. Furthermore, DES gels’ conduction properties may enable their application in biosensors. This study presents an innovative approach to synthesizing polyHEMA gels and provides insights into their potential applications in the biomaterials field.

## 1. Introduction

Poly(2-hydroxyethyl methacrylate) (polyHEMA) hydrogels have emerged as a popular material in biomedical applications, particularly as contact lenses, due to their excellent mechanical properties, biocompatibility, hydrophilicity, and low interfacial tension [1,2]. These hydrogels have been known since the 1960s and were the first choice for manufacturing soft contact lenses based on hydrogels. Typically, these hydrogels are prepared by co-polymerization of HEMA and a crosslinking agent in an aqueous solvent, resulting in a three-dimensional network composed mostly of water [3,4]. However, their high susceptibility to evaporation in ambient environments causes dehydration, ultimately altering their properties [5,6]. Additionally, the heterogeneous microstructures formed due to randomly distributed crosslinking points in chemically crosslinked hydrogels lead to non-uniform energy dissipation and poor mechanical properties [7,8,9]. Therefore, there is a pressing need to develop polyHEMA with low dehydration tendencies and high mechanical properties for superior performance.

One promising approach is to use deep eutectic solvents (DES) in gel preparation instead of aqueous solutions. DES-based gels have a range of beneficial properties, such as low volatility, high stretchability, and ionic conductivity [10,11,12,13]. For instance, Lai et al. reported a cellulose nanocrystal ionogel with low volatility [11], and Qin et al. prepared transparent, stretchable, and ionically conductive gels by mixing gelatin into a DES for skin devices [12]. DES is typically formed by mixing salt, such as ammonium or phosphonium, with a hydrogen bond donor, such as glycerol, urea, or citric acid, to achieve a low-melting eutectic mixture. As a result, DES possesses unique physicochemical properties, such as an ion-dense nature, excellent solvent properties, and a low volatility [14,15]. DES gels have a uniform polymer network and exhibit higher elasticity compared to gels prepared using other solvents [12,16]. Furthermore, DES is an inexpensive and environmentally friendly alternative to other solvents, making it an effective solution to address the dehydration and strength issues of hydrogels [17,18,19].

When designing and manufacturing polyHEMA for contact lenses, transparency is another critical consideration. Bulk polymerization of polyHEMA hydrogel-based contact lenses frequently results in poor optical transmittance [20,21]. Therefore, incorporating other hydrophilic materials, such as methacrylic acid, N-vinyl-2-pyrrolidone, acrylamide, or sulfobetaine methacrylate, into the polyHEMA network is a common strategy to enhance their performance [21,22,23]. Nevertheless, the high water content alters the microstructural environment of the polymer network and diminishes its mechanical properties [21,24]. Therefore, the development of transparent hydrogels is a significant concern for medical equipment manufacturers.

In this study, we aimed to address the aforementioned challenges by utilizing deep eutectic solvents (DES) as a medium for HEMA polymerization. We detail the synthesis of polyHEMA networks in both deionized (DI) water and a DES-based on choline chloride (ChCl) and ethylene glycol (EG) in a 1:1 molar ratio of ChCl:EG and investigate the impact of the solvents on the properties of the resulting polymeric materials. Our findings show that the polymerization process in DES occurred more rapidly than in DI water, and the resulting polyHEMA DES gels exhibited enhanced stretchability, nonvolatility, and optical transparency compared to those formed in DI water. Furthermore, polyHEMA DES gels displayed conductive properties. To the best of our knowledge, this study represents the first attempt to synthesize a contact lens using DES as a base, which holds great potential for development in the field of wearable sensors.

## 2. Experimental Method

### 2.1. Materials

2-Hydroxyethyl methacrylate (HEMA 97%), ethylene glycol dimethacrylate (EGDMA, 98%), and ammonium persulfate (APS, 98%) were purchased from ACROS (Geel, Belgium). Choline chloride (ChCl, 98%) was purchased from Alfa Aesar (Ward Hill, MA, USA). Ethylene glycol (EG, 99%) was obtained from Showa Chemical (Tokyo, Japan). Milli-Q gradient water, with a resistivity of 18.2 MΩ·cm, was utilized as the deionized water (DI water) in the experiments.

### 2.2. Preparation of PolyHEMA DES Gels

Four DES gels with various HEMA contents were prepared. The compositions of the gels are listed in Table 1. The DES only functions as a solvent to dissolve the monomers for a homogeneous polymerization [14]. First, a DES was prepared by stirring a mixture of ChCl and EG in a 1:1 molar ratio at 100 °C for 30 min until a homogeneous colorless liquid was formed. Next, HEMA and EGDMA were mixed into the DES. After uniformly mixing all components, an APS aqueous solution was added to initiate polymerization. Each mixture was immediately poured into a contact lens mold or into two poly(tetrafluoroethylene) (PTFE) plates separated by a 2 mm thick spacer. After 24 h of polymerization at 70 °C, the solids (i.e., DES gels) were obtained.

### 2.3. Preparation of polyHEMA Hydrogels

PolyHEMA hydrogels using deionized (DI) water as the solvent were synthesized as control groups. First, HEMA and EGDMA were dissolved in DI water, followed by the addition of APS to initiate polymerization. Each mixture was immediately poured into a contact lens mold or between two PTFE plates separated by a 2 mm thick spacer. After 24 h of polymerization at 70 °C, the solids were immersed in water several times to remove residual unreacted agents and obtain the hydrogels. The compositions of hydrogels are listed in Table 1.

### 2.4. Characterization

The chemical structures of the DES gels and hydrogels were identified by Fourier transform infrared (FTIR) spectroscopy (Spectrum One, PerkinElmer) using an attenuated total reflection (ATR) diamond crystal accessory. FTIR spectra were scanned in the absorbance mode from 4000 to 650 cm^−1^ at a resolution of 4 cm^−1^, and 48 accumulation scans were collected.

The transmittance of the DES gels was measured using a UV-visible spectrophotometer (ChromTech CT-2800, Taiwan). Each hydrogel was mounted in a quartz cuvette, and the spectrum was scanned at wavelengths ranging from 380 to 780 nm [25].

For the swelling test, the initial weight of the test pieces was measured after the crosslinking process. Subsequently, the test pieces were immersed in either DES or DI water at room temperature until reaching a state of equilibrium. Afterward, the test pieces were reweighed. The swelling ratio (Q) was calculated as (ms − mi)/mi, where ms represents the weight of the swollen specimen and mi denotes the weight of the initial specimen.

A texture analyzer (EZ-SX, Shimadzu) with a 500 N load cell was used to measure the compressive and tensile moduli of various DES gels and hydrogels. The compressive test was performed on 2 mm thick samples, which were cut into 5 mm diameter pieces and placed on the stage at room temperature [26]. For the tensile test, 1 mm thick samples were cut into 3 cm × 0.5 cm (length × width) pieces and mounted in a tension clamp at room temperature [25]. Both compressive and tensile tests were conducted at a rate of 1 cm/min. The compressive and tensile modulus values were calculated as the slopes of the best-fit lines from the linear region of the measured stress–strain data in the low-strain regime (<40%). To obtain average values, each measurement was repeated at least three times.

In the dehydration test, the weight of fully swollen gel disks, with a diameter of 5 mm and a thickness of 2 mm, was measured initially. These gel disks were then exposed to ambient conditions at 25 °C and 64% humidity for a specific duration. After dehydration, the disks were reweighed to determine their new weight. The remaining weight fraction of the gel disks was calculated using the equation: (md/ms) × 100 (%), where md represents the mass of the dehydrated gel disk and ms represents the mass of the fully swollen gel disk.

### 2.5. Statistical Analysis

The statistical analysis for different groups was conducted using the Student’s *t*-test. The reported data include mean values along with the corresponding standard deviation (SD). The statistical significance threshold was set at *p* ≤ 0.05. The GraphPad Instat 3.0 program (GraphPad Software, San Diego, CA, USA) was used for all statistical analyses.

## 3. Results and Discussion

### 3.1. Transmittance

**Figure 1:**
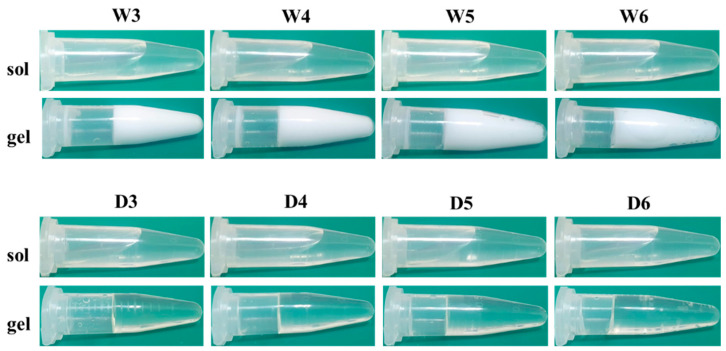
Visual representation of polyHEMA network prepared with water and DES and their sol-gel transitions.

**Figure 2:**
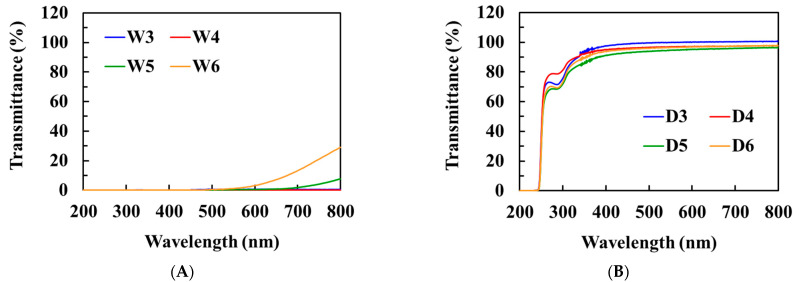
UV-vis spectrum of hydrogels (**A**) and DES gels (**B**) with different weight ratios of HEMA.

Initially, we synthesized a series of polyHEMA DES gels with different mass ratios of HEMA using a DES, while polyHEMA hydrogels (prepared with DI water) were used as control groups. As shown in Figure 1, before crosslinking, HEMA solutions in DI water, as well as DES, were transparent, indicating that the monomers were homogeneously dissolved in both solvents. No HEMA/water or HEMA/DES solubility issues were observed, even though the concentration of HEMA was increased up to ~50% (*v*/*v*). After cross-linking, the W3, W4, W5, and W6 hydrogels became opaque when water was used as the solvent. This opacity resulted from phase separation, which caused the formation of heterogeneous microstructures [27]. In contrast, polymerization of the D3, D4, D5, and D6 DES gels led to the formation of transparent gels, indicating that DES is a good solvent system for HEMA and polyHEMA. The transparency of the D3, D4, D5, and D6 DES gels was further measured by scanning with a UV-vis spectrophotometer in the wavelength range of 380–780 nm (Figure 2). All DES gels exhibited a transmittance of over 95%. It is noteworthy that, according to the International Organization for Standardization of Contact Lens (ISO 18369), materials possessing a transmittance greater than 90% are considered adequately transparent for ophthalmic applications [25].

### 3.2. Study of Polymerization Kinetics by FTIR Analysis

**Figure 3:**
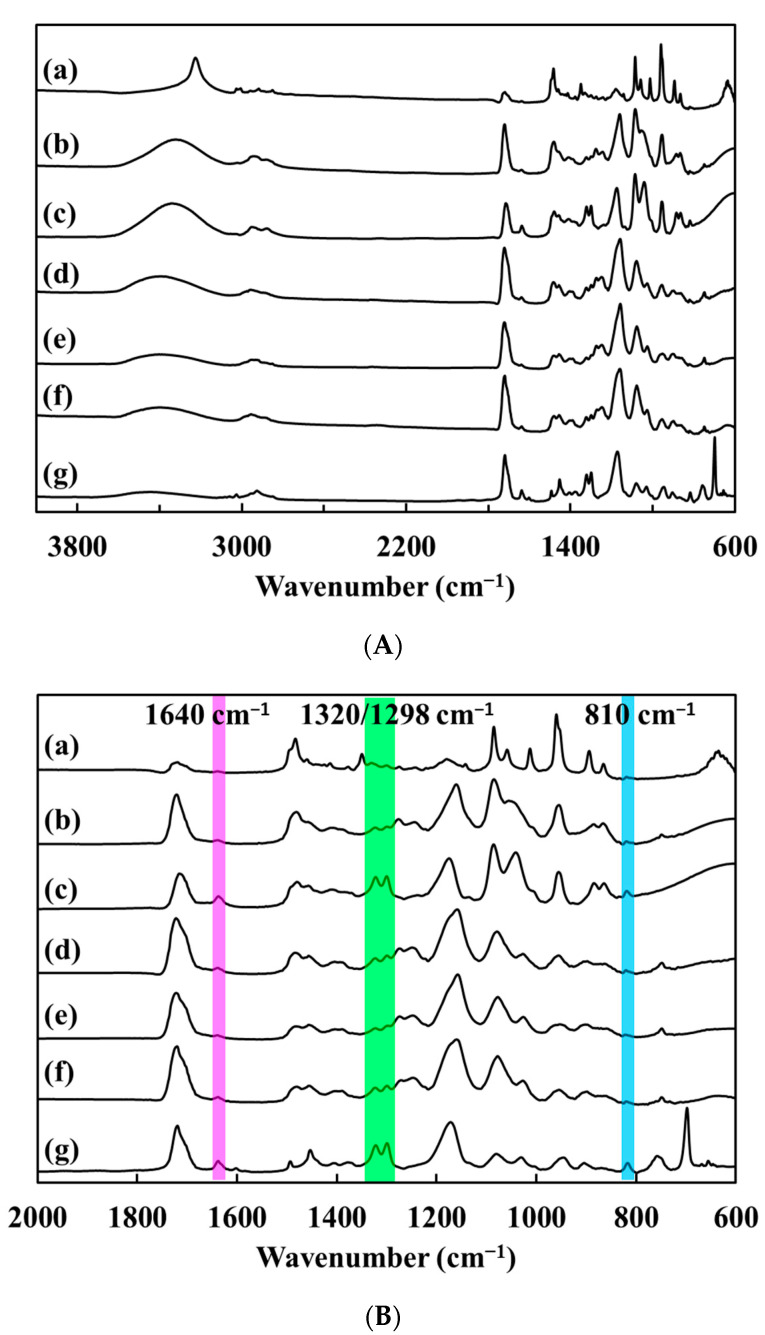
ATR-FTIR spectra (**A**) and enlarged wavenumber at 2000–600 cm^−1^ of a polyHEMA hydrogel (a to c), a DES gel (d to f), and a HEMA/PEGDA co-monomer mixture (g). The vertical dashed line indicates the position of the peaks associated with the polymerizable methacrylate/acrylate group. (**B**) The peaks disappeared as the reaction time increased, indicating complete copolymerization. Polymerization time: 1.5 h (c & f); 6 h (b & e); 24 h (a & d).

ATR-FTIR analyses were conducted to verify the complete copolymerization of HEMA and EGDMA within the DES gel samples. As shown in Figure 3, the spectrum of the unreacted HEMA/EGDMA mixture (45% HEMA with 1% EGDMA) has peaked at 1298 cm^−1^ and 1320 cm^−1^, attributed to vinyl group (CH_2_=CH–) deformation/wagging, and the peaks observed at 810 and 1640 cm^−1^ are due to vinyl group (CH_2_=CH–) bending and C=C stretching vibrations, respectively [28]. After 1.5 h of reaction, the peaks associated with the HEMA and EGDMA precursors disappeared from the DES gel spectrum but not from the hydrogel spectrum, indicating that HEMA and EGDMA were copolymerized in the DES gel sample, but the hydrogel sample had not yet fully copolymerized. After 6 h of reaction, the peaks associated with the HEMA and EGDMA precursors disappeared from the hydrogel spectrum. Similar findings have been reported in a previous study on poly(N-isopropylacrylamide) hydrogels synthesized via free radical polymerization in deep eutectic solvents [29]. Bednarz et al. also investigated the formation of a poly(itaconic acid) network in both DI water and DES, noting that the critical monomer conversion at the gel point was higher in DES compared to the water [30]. This suggests that the polymerization rate within DES was faster than that within DI water. Another study utilized a DES composed of choline chloride and urea as a solvent for reversible addition-fragmentation chain-transfer (RAFT) polymerization of HEMA. Notably, the conversion of HEMA was significantly enhanced to 91% when compared to only 71% achieved using dimethylformamide (DMF) as the solvent [31]. The accelerated polymerization rate in DES was attributed to the polar, ionic, and hydrogen bonding interactions between the monomer and the DES solvent [29,31].

### 3.3. Swelling Properties

The swelling property of a gel refers to its ability to absorb and retain solvent within its network structure. When a gel undergoes swelling, its volume increases as the solvent fills the gaps between polymer chains. A significant degree of swelling indicates a higher capacity for solvent absorption, suggesting a lower crosslinking density. Conversely, a lower degree of swelling implies a tighter network structure with a higher crosslinking density [32,33]. In this study, both DES gels and hydrogels were prepared by fixing the crosslinker amount and varying the HEMA monomer concentration. It can be anticipated that with an increase in the monomer concentration, the swelling degree is greater, indicating a lower crosslinking density (Table 1). By comparing the preparation of equivalent amounts of HEMA in DES and DI water, it was observed that the swelling of DES gels was lower than that of hydrogels, indicating a higher crosslinking density in DES gels.

### 3.4. Mechanical Properties

**Figure 4:**
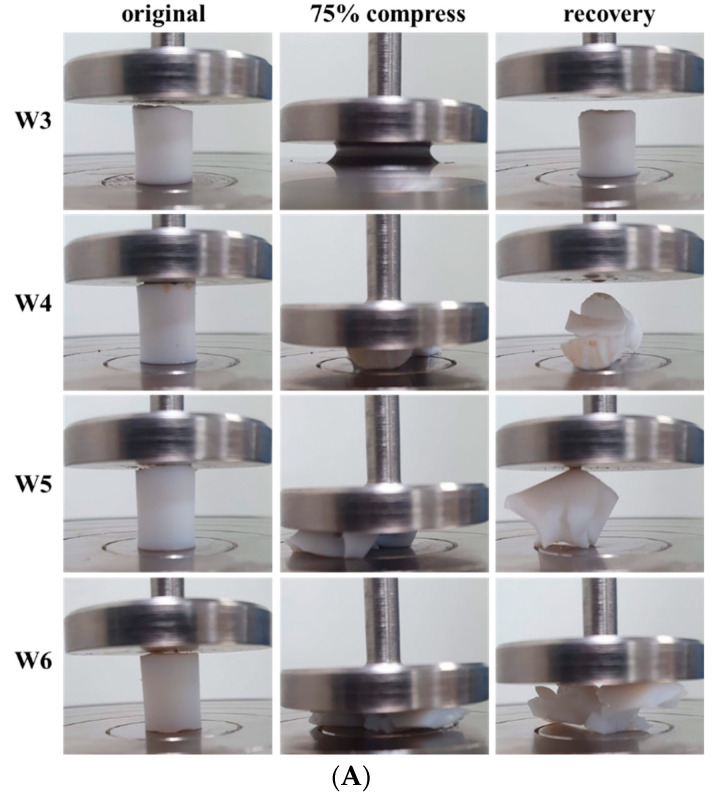

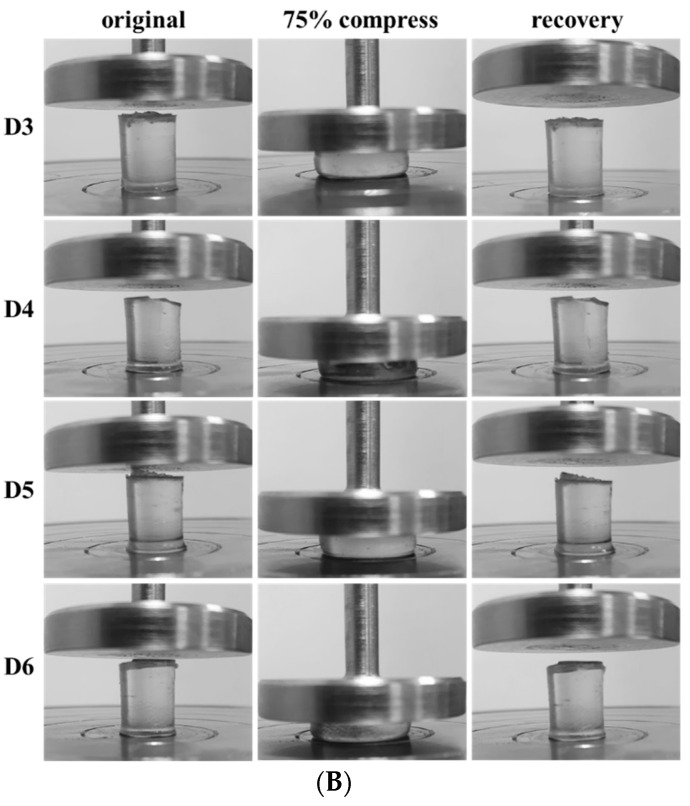
Images of fragile hydrogels (**A**) and tough DES gels (**B**) before and after 75% compression.

**Figure 5:**
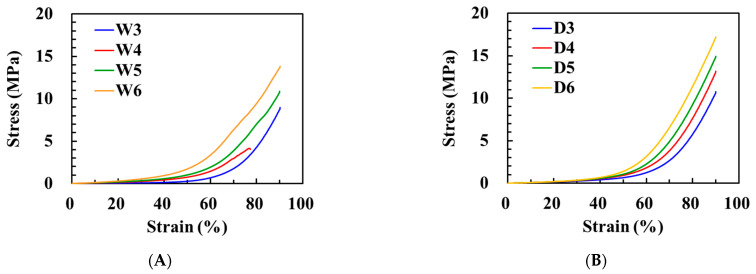

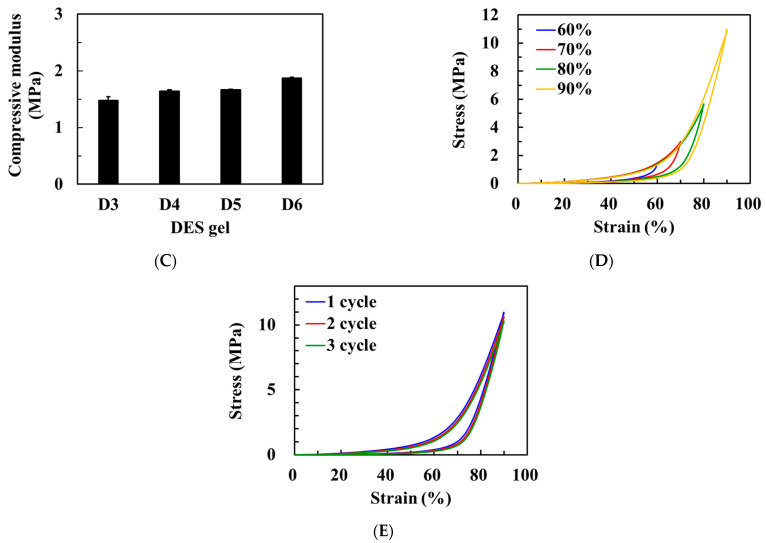
Compressive stress−strain curves of polyHEMA hydrogels (**A**) and DES gels (**B**) with different weight ratios of HEMA. (**C**) Young’s moduli of the DES gels with different weight ratios of HEMA. (**D**) Compressive stress−strain curves of D5 gel with varying maximum compression under loading–unloading cycles. (**E**) Compressive stress–strain curves of D5 gel at 90% strain for three loading–unloading cycles.

The high toughness of the polyHEMA DES gels was observed through a compressive test. Under compressive loading, polyHEMA hydrogels with only covalent crosslinking exhibited dramatic and sudden failure, especially W4, W5, and W6, while W3 exhibited ductile and unrecoverable failure (Figure 4A). Conversely, all DES gels demonstrated the ability to withstand compression without incurring irreversible damage, which can be attributed to the interaction between the -OH functional group of HEMA and the Cl anion through the hydrogen bonding [28]. The DES gels recovered to their original cylindrical shape immediately after releasing the pressure (Figure 4B). Compressive stress–strain curves for both hydrogels and DES gels with varying HEMA contents are shown in Figure 5A,B, respectively. As shown in Figure 5A, the W3 hydrogels did not reach breaking point when the hydrogels were compressed at 90% strain. However, breaking was observed at 70% strain for the W4, W5, and W6 hydrogels due to the permanent rupture of covalent bonds. The fracture stresses of the W4, W5, and W6 hydrogels were 2.85, 3.25, and 5.66 MPa, respectively.

In contrast, the DES gels demonstrated an impressive capacity to withstand compression up to 90% strain without fracturing due to the presence of secondary crosslinking (i.e., hydrogen bonding) within their polymeric networks (Figure 5B) [34,35]. The compressive modulus of the DES gels increased with an increase in the HEMA content (Figure 5C). Specifically, the compressive modulus values for D3, D4, D5, and D6 gels were recorded as 1.48, 1.64, 1.67, and 2.30 MPa, respectively. Additionally, the DES gels exhibited rapid recovery and excellent fatigue resistance properties. Figure 5D demonstrates the ability of the D5 gel to fully recover its initial state even under a high strain of 90%. Furthermore, three continuous compression–relaxation cycles were applied to test the recovery of the D5 gel, and the stress–strain curves for all cycles were nearly identical, indicating substantial removal of the plastic deformation [12,36].

**Figure 6:**
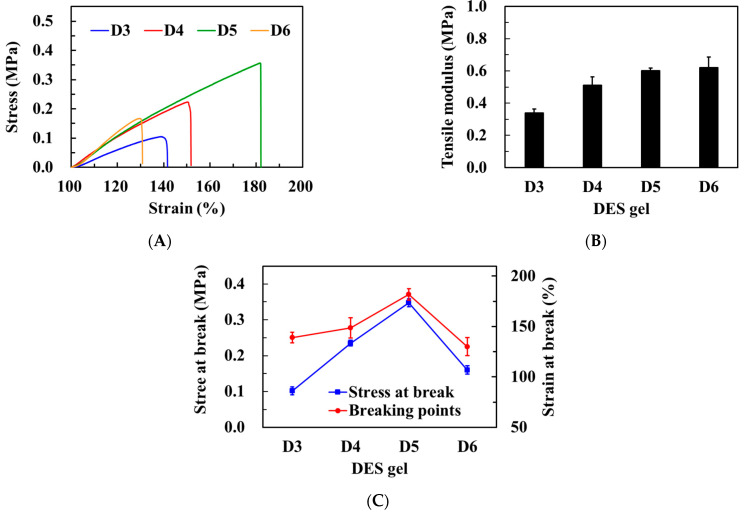
(**A**) Tensile stress−strain curves of DES gels with different weight ratios of HEMA. (**B**) Young’s moduli of DES gels with different weight ratios of HEMA. (**C**) Relationship of the stress and strain at break values with the different weight ratios of HEMA in DES gels.

The strength of DES gels was further assessed through a tensile test, and the results are presented in Figure 6A. It was observed that the stress at break increased significantly from 0.10 to 0.35 MPa, and the strain at break also increased significantly from 139% to 182% as the concentration of HEMA increased from 33% to 45%. However, at a HEMA content of 49%, the stress and strain at break decreased to only 0.16 MPa and 130 %, respectively (Figure 6C). This suggests that the DES gels became stiffer and more brittle at excessively high HEMA concentrations, as observed in the D6 gel. It is speculated that when the HEMA concentration exceeds the solubility limit of DES, uneven interactions occur between HEMA and DES during the polymerization process. As a result, stronger solvent–solvent or polymer–polymer interactions occur compared to interactions between the polymer and solvent components, leading to non-uniform force dispersion and ultimately causing gel damage under the tension [37]. Additionally, Young’s moduli of the DES gels were in the range of 0.3 to 0.65 MPa, and the tensile modulus significantly increased with an increase in HEMA concentration (Figure 6B). Soft contact lenses with moduli ranging from 0.3 to 1.52 MPa are considered comfortable for the wearer [38]. Based on the compressive and tensile test results, it is believed that the mechanical properties of the D5 gel are similar to those of soft contact lenses.

### 3.5. Dehydration

**Figure 7:**
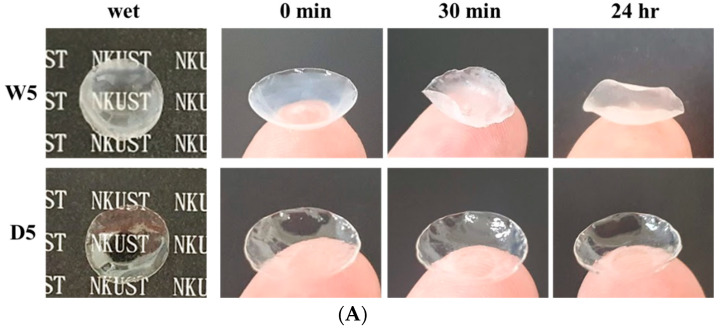

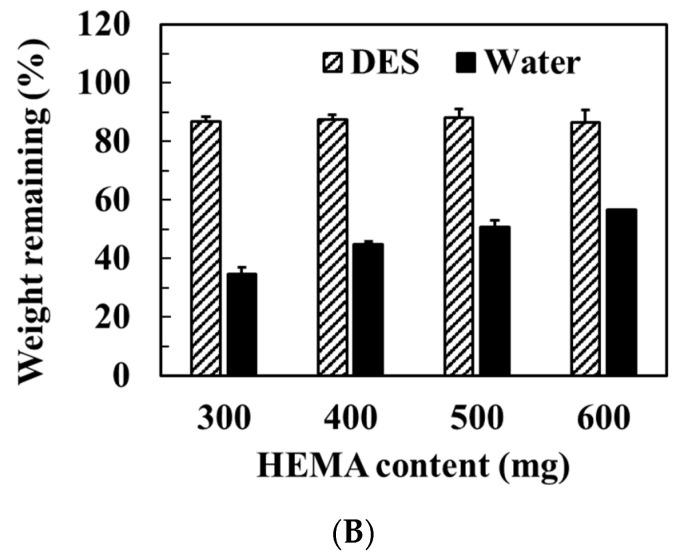
(**A**) Photographs of contact lenses prepared using the W5 hydrogel and the D5 gel before and after 24 h in air. (**B**) Remaining weight of W5 hydrogel and D5 gel was in a constant humidity (64%) and constant temperature (25 °C) environment for 24 h.

The use of DES gel over hydrogel has the added benefit of having an extremely low volatility [12], as demonstrated by Figure 7A,B. Figure 7A shows that the DES gels retained their shape even after being stored under ambient conditions for 24 h, while the hydrogels experienced deformation within just 30 min due to water evaporation. The hydrogels also lost approximately half of their original weight after 24 h, indicating significant water loss. In contrast, the DES gels only experienced a minor weight loss of approximately 10% (Figure 7B), providing evidence of the excellent non-volatility and compatibility between DES and the polymer network. Recent studies have shown that DES gels have good biocompatibility and hemocompatibility, making them suitable for use in biomedical materials and tissue engineering applications [39,40]. As a result, we believe that DES gels have significant potential for use in soft contact lenses in the future.

### 3.6. Conductivity

**Figure 8:**
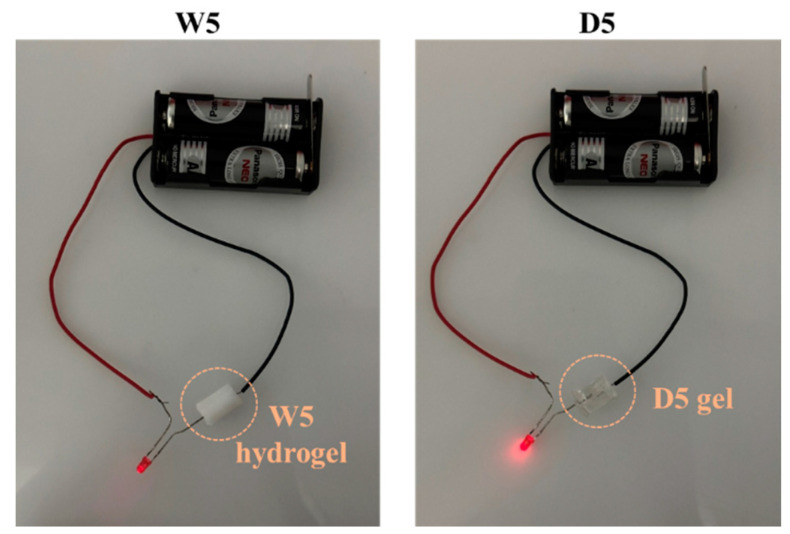
LED emission testing in an electrical circuit connected in series with W5 hydrogel and D5 gel.

DES is formed by mixing a hydrogen bond acceptor (HBA) and a hydrogen bond donor (HBD) in specific molar ratios, which creates a low melting point mixture that is lower than that of each individual component. The presence of HBA and HBD in DES allows for the formation of charged species, such as protonated HBDs or deprotonated HBAs, contributing to the overall conductivity of the system [11,13]. By incorporating DES as a solvent in the synthesis of gels, it becomes possible to imbue them with electrical conductivity. This is because the charged species from DES can become integrated into the gel network, creating a conductive path. As experiments had shown, while the W5 hydrogel failed to emit light when a LED was embedded, the D5 gel displayed a bright LED, indicating excellent electrical conductivity (Figure 8). The DES-based polyHEMA with electrical conductivity has the potential to revolutionize the development of sensitive and high-performance biosensors for use in biomedical applications [41].

## 4. Conclusions

The present study involved the synthesis of a polyHEMA gel using a DES mixture of choline chloride and ethylene glycol (in a 1:1 molar ratio). A comparison was made between the polymerization kinetics of HEMA in water and DES, revealing a faster conversion of HEMA in the DES medium. The DES gels exhibited superior characteristics, such as higher transparency, increased crosslinking density, enhanced toughness, reduced dehydration, and improved conductivity compared to conventional hydrogels. Furthermore, the influence of varying HEMA concentrations on the compressive and tensile properties of the DES gels was investigated. It was observed that as the HEMA concentration increased, the compressive and tensile moduli of the DES gels also increased. Notably, the gel with 45% HEMA content demonstrated remarkable compression–relaxation cycles and exhibited the highest strain at the point of rupture in the tensile test. However, when the HEMA concentration exceeded 45%, the DES gel became rigid and brittle, likely due to uneven interactions between HEMA-DES, HEMA-HEMA, and DES-DES. These findings highlight the potential of DES in the manufacturing of contact lenses, while the additional conductivity feature opens up possibilities for developing stimulus-responsive intelligent hydrogels for wearable sensing applications.

## Figures and Tables

**Table 1:** Feed composition and swelling property of samples.

Samples	HEMA (mg)	EGDMA (μL)	APS ^a^ (μL)	H_2_O (μL)	DES ^b^ (μL)	Swelling (Q)
W3	300	10	20	600	-	0.102 ± 0.031
W4	400	10	20	600	-	0.171 ± 0.053
W5	500	10	20	600	-	0.271 ± 0.075
W6	600	10	20	600	-	0.442 ± 0.085
D3	300	10	20	-	600	0.283 ± 0.084
D4	400	10	20	-	600	0.342 ± 0.059
D5	500	10	20	-	600	0.374 ± 0.079
D6	600	10	20	-	600	0.440 ± 0.062

^a^ 10% APS was dissolved in DI water. ^b^ DES was prepared by stirring a mixture of ChCl and EG in a 1:1 molar ratio.

## Data Availability

Data will be made available on request.
